# A 2-Year Longitudinal Seroepidemiological Evaluation of *Toxoplasma gondii* Antibodies in a Cohort of Autochthonous Sheep from Central Portugal

**DOI:** 10.3390/pathogens10010040

**Published:** 2021-01-06

**Authors:** Daniela Almeida, João Quirino, Patrícia Ferreira Barradas, Priscilla Gomes da Silva, Maria Pereira, Rita Cruz, Carla Santos, Ana Cristina Mega, Fernando Esteves, Carmen Nóbrega, Helena Vala, Fátima Gärtner, Irina Amorim, João R. Mesquita

**Affiliations:** 1 Institute of Biomedical Sciences Abel Salazar (ICBAS), University of Porto, 4050-313 Porto, Portugal; dgomesalmeida23@gmail.com (D.A.); jp_quirino93@hotmail.com (J.Q.); patriciaferreirabarradas@gmail.com (P.F.B.); priscilla@ua.pt (P.G.d.S.); fgartner@ipatimup.pt (F.G.); iamorim@ipatimup.pt (I.A.); 2 Epidemiology Research Unit (EPIUnit), Instituto de Saúde Pública, University of Porto, 4050-091 Porto, Portugal; 3 Agrarian School of Viseu, Polytechnic Institute of Viseu (ESAV), 3500-606 Viseu, Portugal; mapereira@esav.ipv.pt (M.P.); rcpaiva@esav.ipv.pt (R.C.); casarede@esav.ipv.pt (C.S.); amega@esav.ipv.pt (A.C.M.); festeves@esav.ipv.pt (F.E.); cnobrega@esav.ipv.pt (C.N.); hvala@esav.ipv.pt (H.V.); 4 Global Health and Tropical Medicine, Instituto de Higiene e Medicina Tropical, 1349-008 Lisboa, Portugal; 5 Centre for Studies in Education and Health Technologies (CI&DETS), 3500-606 Viseu, Portugal; 6 Centre for the Research and Technology of Agro-Environmental and Biological Sciences (CITAB), University of Trás-os-Montes e Alto Douro, 5001-801 Vila Real, Portugal; 7 Institute of Molecular Pathology and Immunology, University of Porto (IPATIMUP), 4099-002 Porto, Portugal; 8 Institute for Research and Innovation in Health (i3S), University of Porto, 4099-002 Porto, Portugal

**Keywords:** *Toxoplasma gondii*, sheep, epidemiology, serology

## Abstract

(1) Background: *Toxoplasma gondii* is an important zoonosis and one of the major causes of abortion in sheep worldwide. (2) Methods: We performed a 2-year longitudinal serological anti-*T. gondii* IgG screening on a cohort of a spatially confined population of a Portuguese autochthonous sheep breed in central Portugal. (3) Results: From the screening of the 2015 and 2016 sera, an increase of seroprevalence was observed (57.7% (95% CI: 49.9–65.3%) versus 69.1% (95% CI: 61.5–75.9), from 2015 and 2016, respectively) (*p* = 0.031). (4) Conclusions The present study is the first to provide prospective data on the anti-*T. gondii* serological status of a sheep cohort in Portugal, showing an increase in the occurrence of *T. gondii*. There is a need to provide a clearer understanding of *T. gondii* epidemiology in Portugal, ideally by implementing monitoring programs on sentinel herds, not only due to the high impact of *T. gondii* on animal health but also for it being a zoonosis.

## 1. Introduction

*Toxoplasma gondii* is a zoonotic intracellular protozoan with a complex heteroxenous lifecycle that has a worldwide distribution [1]. Felids are the definitive hosts of *T. gondii,* where sexual reproduction of the parasite occurs, with asexual reproduction occurring in intermediate hosts that are warm-blooded animals (including humans) [1,2,3]. Toxoplasmosis is mainly transmitted by the ingestion of cysts present in undercooked or raw meat and contaminated water or food, through sporulated oocysts or by congenital transmission [1,2,3]. Human serosurveillance studies performed worldwide on specific anti-*T. gondii* IgG showed that seroprevalences in humans vary between 1% and 100%, being dependent on several variables such as socioeconomic and environmental circumstances, including eating habits, hygiene, susceptibility of hosts, location, and soil humidity [1,4,5,6]. A higher number of infections is observed with increasing age, and in humid/warm climates [4]. Even though infection by *T. gondii* is usually asymptomatic in humans, it may cause fatal disorders in immunosuppressed people and pregnant women, justifying the public health importance of this parasite [1]. Sheep are usually infected by ingestion of sporulated oocysts in contaminated feed or pasture, which then excyst in the small intestine, invade the intestinal mucosa, and multiply as tachyzoites in regional lymph nodes. Hematogenous spread and the subsequent immune response either clear the tachyzoites or transform them into bradyzoites that are sequestered in tissue cysts in many edible parts of the animal [7]. The naive and pregnant ewe is susceptible to toxoplasma-induced reproductive losses when tachyzoites invade the uterus and placenta, mainly causing reproductive disorders such as abortion, embryonic reabsorption, fetal mummification, and stillborn and congenital malformations of the fetus. [8]. Noteworthy, toxoplasmosis is considered to be one of the most important differential diagnosis for abortion in sheep. Abortions will only occur in susceptible animals, mainly in those infected during the initial stages of pregnancy. However, a variety of outcomes can occur if oocysts are ingested at an advanced stage of gestation, with vertical transmission generating births of weak or even normal lambs [7]. 

In Portugal, to the best of our knowledge, only two studies have been completed evaluating the presence of antibodies anti-*T. gondii* in sheep, both focusing on the north region of Portugal [9,10]. A 2009 study from the north region of the country detected 17.1% of sheep as seropositive by a microscopic agglutination test (MAT), suggesting that *T. gondii* played an important role in sheep abortions and neurological signs in lambs [9]. A later study (2013) also in the northern region of the territory showed that 33.6% of the sampled sheep were positive for IgG anti-*T. gondii,* also by MAT. These studies seem to be in agreement with those published in other Mediterranean countries showing IgG seroprevalences in 40.1% of ruminants in Cyprus [11], 46.5% of sheep in southern Spain [3], and 28.6% of sheep in Italy [12].

To study the seroprevalence and detailed spatial distribution of *T. gondii* associated with sheep in central Portugal, we performed a 2-year longitudinal serological anti-*T. gondii* screening on a cohort of a spatially confined population of a Portuguese autochthonous sheep breed.

## 2. Experimental Section

### 2.1. Geographic, Climatic, and Husbandry Contexts

The study included a confined population of a Portuguese autochthonous sheep breed (Serra da Estrela), to best reflect the circulation of *T. gondii* in this region. Serra da Estrela sheep are reared solely in the Serra da Estrela mountain (elevation 1993 m), the highest mountain of mainland Portugal, located at the central region of the country. This sheep breed is used to produce milk for making cheese of the brand “Serra da Estrela”, an artisanal “Protected Designation of Origin” (PDO) product, highly valued both in the country and internationally. The region is considered a semi-natural Mediterranean pasture, consisting of rugged and mountainous regions, composed of shrub and herbaceous strata, usually associated with the grazing of the sheep. The warmest month is July and the coldest is January, with an average annual temperature lower than 7 °C, mostly in the plateau areas. Average precipitation values vary between 1000 mm in the territories of the Mondego valley, Seia, and Gouveia and values above 2500 mm per year at the highest altitudes of the central plateau. Despite its irregular pattern, rainfall occurs mainly between November and March. The National Association of Serra da Estrela Sheep Breed—ANCOSE—manages the autochthonous Serra da Estrela sheep, a breed with legislated protected origin that is solely bred in this region, with animals having little or no movement, thus providing a viable and detailed tool for animal disease surveillance in this region [13]. Only the Serra da Estrela autochthonous breed was selected for this study, given its geographical restriction to the region and mobility restraints (sheep are housed during the night and are allowed movement uniquely on the farm premises). Breeding of further domestic animal species is residual in this region and there is evidence for circulation of wild game in the territory [13]. 

### 2.2. Longitudinal Anti-T. gondii Serosurvey

Sera collected from a previous study was used [14]. Briefly, blood from 168 “Serra da Estrela” breed sheep was collected in January/February 2015, and again in January/February 2016 from the same animals (168 paired samples, for a total of 336). These 168 sheep were considered representative of the Serra da Estrela sheep population, as confirmed by sample size calculation (population size of 70,000, expected seroprevalence of 17% [9], absolute error of 6%, 95% confidence level, and 40% oversampling to account for deaths, sales, and exchanges [15] were considered). A random selection of four sheep (aged more than 6 months; circa 10% of average herd size) was performed on each of the 42 “Serra da Estrela” sheep herds belonging on 9 municipalities of the region (Arganil, Carregal do Sal, Celorico da Beira, Fornos de Algodres, Gouveia, Mangualde, Oliveira do Hospital, Seia, Tábua). None of the sheep were vaccinated for toxoplasmosis and farmers of the sheep included in the study reported that they do not control stray or domestic cat movement. All 336 sera were analyzed individually against *T. gondii* IgG antibodies, using a commercial enzyme-linked immunosorbent assay (ELISA ID Screen Toxoplasmosis Indirect Multi-Species Kit, IDvet, Grabels, France). This assay is an indirect ELISA using P30 antigen of *T. gondii* for coating microwells. Procedures were performed according to the manufacturer’s instructions. Optical densities (OD) were read at 450 nm and the results were evaluated by calculating the sample to positive ratio (S/P%). Samples with a S/P% ≤ 40% were considered negative and samples with a S/P%≥ 50% were considered positive. Values ranging between 40% and 50% were considered doubtful. If the result was again doubtful after repeating the test, samples were considered negative. The chi-square test was used to study differences between the occurrences found in years 2015 and 2016 (GraphPad Prism version 5.04), and a *p* value < 0.05 was considered statistically significant.

## 3. Results

Results from the serosurvey show that anti-*T. gondii* IgG antibodies were found in both years and no sera provided doubtful results. Of the 168 sera samples from the 2015 sample collection, 97 tested positive for IgG anti-*T. gondii*, corresponding to a seroprevalence of 57.7% (95% confidence interval (CI): 49.9–65.3%). From the total of 168 sera of the 2016 sample collection, 116 tested positive for IgG anti-*T. gondii*, showing a 69.1% (95% CI: 61.5–75.9) seroprevalence. The occurrence of seropositive animals in 2015 and 2016 is detailed on Table 1. 

Anti-*T. gondii* seroprevalence differences in the 2015 and 2016 samplings were statistically significant (*p* = 0.031). When studying seroprevalences within each municipality, statistically significant differences were only found in Oliveira do Hospital. In this municipality, from the 2015 collection, 12 out of the 32 animals were positive for IgG anti-*T. gondii* (37.5%; 95% CI: 21.1–56.3). When again tested in 2016, 20 out of the same 32 animals tested positive (62.5%; 95% CI: 43.7–78.9) (*p* = 0.046).

From the 97 animals that tested positive for the presence of IgG anti-*T. gondii* in 2015, 6 became seronegative in 2016 (seroreverted). On the other hand, from the 71 animals initially negative for anti-*T. gondii* IgG, 26 became seropositive in 2016 (seroconverted).

## 4. Discussion

Toxoplasmosis is considered to be one of the most important causes of abortion in sheep [1]. Until today, only two studies have evaluated the circulation and epidemiological features of ovine toxoplasmosis in Portugal by use of serosurveys and both focused on the northern region of Portugal. In the present study we provide the results of a 2-year longitudinal screening on the occurrence of *T. gondii* IgG antibodies in “Serra da Estrela” sheep, a breed that is geographically confined to the central region of Portugal. From the screening of the 2015 and 2016 sera, an increase of seroprevalence was observed (57.7% (95% CI: 49.9–65.3%) versus 69.1% (95% CI: 61.5–75.9), from 2015 and 2016, respectively) (*p* = 0.031). Antibody occurrence seems to be higher than previously reported in other Mediterranean countries during the last years (28.6%-46.5%) [3,11,12]. Anti-*T. gondii* seroconversion was observed in 26 sheep, which was due to exposure/infection by *T. gondii*, showing that an increasing individual risk exists with increased age. This could be in some part explained by the lack of control of free-roaming cats, as reported by farmers. A joint European Food Safety Authority (EFSA) and European Center for Disease Prevention and Control (ECDC) report compiled data from European Union member states and reported that small ruminant toxoplasmosis had decreased in the years of 2015 to 2016 with prevalence dropping by half from 38.8% to 18.7% [16]. The results presented in the present study are contradictory with those from EFSA and may represent an atypical circulation of this agent in this central region of Portugal, supporting the need for more widespread studies across Europe. In the northern region of Portugal, 17.1% and 33.6% of sheep from 2009 and 2013, respectively, were positive by MAT [9,10], which is substantially lower than the prevalence reported in the present study (57.7% and 69.1% in 2015 and 2016, respectively) of sheep from the central region. Caution should be taken when comparing these values since different serological assays were used with different sensitivities/specificities. However, it might be tempting to speculate that these differences might be connected to the different animal production specificities, namely biosecurity/biosafety. The making of the artisanal cheese “Serra de Estrela” requires the use of raw, unpasteurized milk, which can potentially be a vehicle for foodborne *T. gondii* infection. The presence of *T. gondii* DNA in raw sheep and goat milk has been reported [17,18,19] as was the presence of infective *T. gondii* in cow and goat milk cheeses [20,21]. Further studies evaluating the risk for the sheep milk cheese consumer could assist future food safety measures.

## Figures and Tables

**Table 1:** Screening for anti-*T. gondii* IgG antibodies in 168 Serra da Estrela sheep sampled in the years 2015 and 2016 (sampling scheme: four animals/farm).

Municipality	2015 Anti-*T.gondii* Positive/Total: no. (%; CI)	2016 Anti-*T.gondii* Positive/Total: no. (%; CI)	Number of New Infections	*p*
Arganil	2/4 (50%; 6.9–93.2)	3/4 (75%, 19.4–99.4)	1	0.465
Carregal do Sal	11/12 (91.7%; 61.5–99.8)	10/12 (83.3%; 51.6–97.9)	-	0.537
Celorico da Beira	14/32 (43.8%; 26.4–62.3)	20/32 (62.5%; 43.7–78.9)	6	0.133
Fornos de Algodres	2/20 (10%; 1.2–31.7)	5/20 (25%; 8.7–49.1)	3	0.212
Gouveia	14/20 (70%; 45.7–88.1)	16/20 (80%; 56.3–94.2)	2	0.465
Mangualde	5/8 (62.5%; 24.5–91.5)	6/8 (75%; 34.9–96.8)	1	0.590
Oliveira do Hospital	12/32 (37.5%; 21.1–56.3)	20/32 (62.5%; 43.7–78.9)	8	0.046*
Seia	33/36 (91.7%; 77.5–98.3)	34/36 (94.4%; 81.3–99.3)	1	0.643
Tábua	4/4 (100%; 39.8–100)	2/4 (50%; 6.9–93.2)	-	0.103
Total	97/168 (57.7%; 49.9–65.3)	116/168 (69.1%; 61.5–75.9)	22	0.031*

CI, 95% confidence interval; ND, not determined; * *p* value < 0.05.
